# A Giant Reconstruction of α-quartz (0001) Interpreted as Three Domains of Nano Dauphine Twins

**DOI:** 10.1038/srep14545

**Published:** 2015-10-08

**Authors:** S. D. Eder, K. Fladischer, S. R. Yeandel, A. Lelarge, S. C. Parker, E. Søndergård, B. Holst

**Affiliations:** 1 Department of Physics and Technology, University of Bergen, Allegaten 55, 5007 Bergen, Norway; 2 Department of Chemistry, University of Bath, Bath BA2 7AY, United kingdom; 3 Laboratoire Surface et Interface du Verre, UMR 125 CNRS/Saint-Gobain Recherche, 39 Quai Lucien Lefranc, 93303 Aubervilliers Cedex, France

## Abstract

Silica (SiO_2_) is one of the most common materials on Earth. The crystalline form α-quartz is the stable silica polymorph at ambient conditions although metastable forms exist. α-quartz is a piezoelectric material, it can be produced artificially and is widely used for example in electronics and the biosciences. Despite the many application areas, the atomic surface structures of silica polymorphs are neither well understood nor well characterized. Here we present measurements of α-quartz (0001). Helium Atom Scattering combined with Atomic Force Microscopy reveals a giant reconstruction consisting of 5.55 ± 0.07 nm wide ribbons, oriented 10.4° ± 0.8° relative to the bulk unit cell. The ribbons, with the aid of atomistic modelling, can be explained as a self-organised pattern of nano Dauphine twins (nano electrical twins).

α-quartz (low-quartz) is the stable form of silica, one of the most widespread minerals on Earth^1^ and an important industrial material and biological interface^2^. The atomic structure of α-quartz surfaces has been the subject of many theoretical investigations^3^,^4^,^5^,^6^,^7^, there has been significantly less experimental work, presumably due to the particular challenge related to the investigation of insulating surfaces on the atomic/nano scale. Atomic resolution Scanning Tunneling Microscopy (STM) images of thin metal supported silica films were recently published^8^, but there is very little experimental work on the surface of bulk crystals. An early Low Energy Electron Diffraction (LEED) study suggests that all basic planes have (1 × 1) reconstructions^9^. A later paper on the (0001) surface reports a(√84 × √84) R11° diffraction pattern, after heating the crystal above 600 °C^10^. Both LEED experiments were hampered by charging of the surface. A paper from 2007 by authors of this manuscript and coworkers presents measurements which indicate a reconstruction with a periodicity of about 5.7+/–0.15 nm which we were not able to resolve at the time^11^. This periodicity is one of the largest periodicities ever measured on a crystal surface without adsorbates. Other examples of long range reconstruction on oxide crystals can be found in^12^,^13^,^14^.

Here we present a combined theoretical and experimental study of the α-quartz (0001) surface. The xperiments were done using Helium atom scattering (HAS)^15^,^16^ and Atomic Force Microscopy (AFM), the only two methods which can provide strictly surface sensitive information on the atomic/nano scale of bulk insulating materials. Pioneering work on oxide and silicate surfaces using HAS was carried out by Wöll and others^17^,^18^,^19^,^20^.

## Results

### Experiments

**Figure 1:**
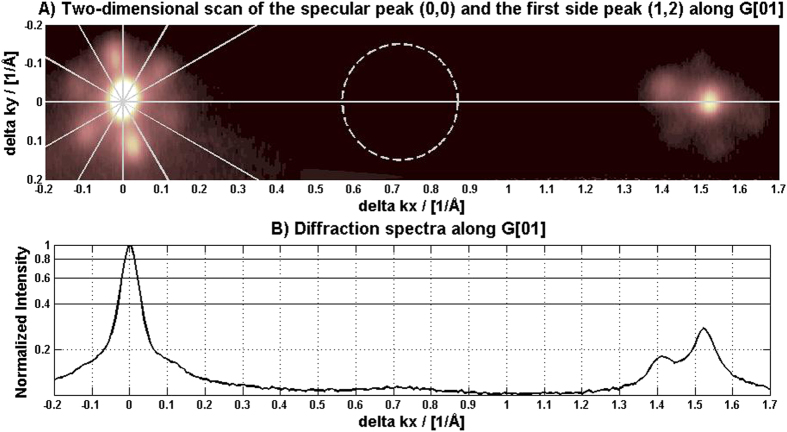
Helium diffraction pattern from the α-quartz (0001) surface. (**A**) 2D pattern along G[01]. The satellite peaks around the specular peak and the first side peak (relative to the bulk structure) are clearly visible. Note the rotation of the satellite peaks relative to the bulk structure. (**B**) 1D scan through the grey line in A, revealing a (weak) (2 × 2) reconstruction peak in the middle.

**Figure 2:**
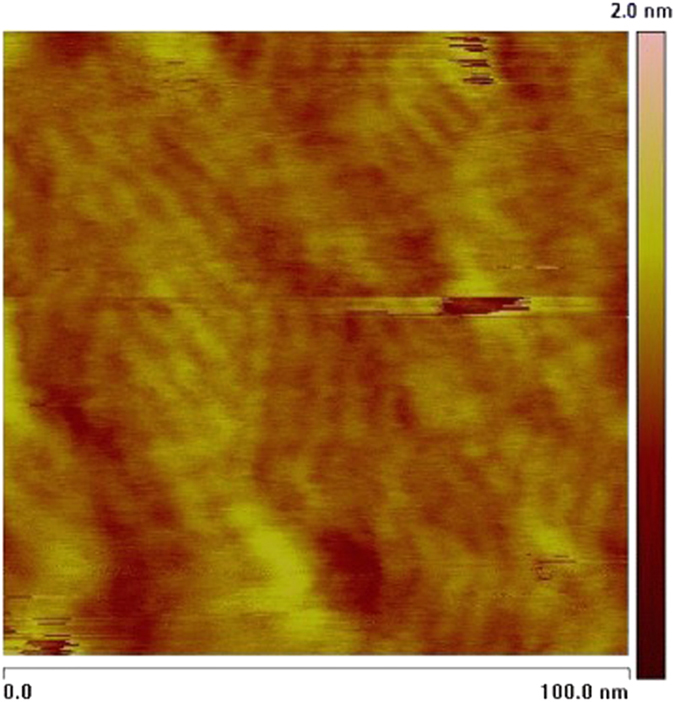
AFM image of an α-quartz (0001) surface. Three domains of “ribbons”rotated 120° relative to each other are clearly visible.

Figure 1 shows the HAS diffraction pattern along G[01]. Rotating the crystal and repeating scans (not shown) revealed a six-fold (2 × 2) pattern with six fold satellite peaks. The satellite peaks have a periodicity of 5.55 ± 0.07 nm, rotated 10.4° ± 0.8° relative to the bulk unit cell. For a hexagonal surface a (2 × 2) reconstruction pattern cannot be distinguished from a pattern stemming from three (2 × 1) domains rotated 120° relative to each other. Figure 2 shows an AFM image of the α-quartz (0001) surface, clearly displaying three domains rotated 120° relative to each other. Each domain area is approximately rhombus shaped, with each rhombus having a width of a few tens of nanometers (there is some size variation). On the basis of the AFM images we can conclude that the diffraction pattern stems from three domains. To account for the satellite peaks in the diffraction pattern each domain area must have a long range periodic structure (“ribbons”) in addition to the (2 × 1) structure. Going back to the AFM image in Fig. 2 we can see that within each domain area a periodic structure of “ribbons” is clearly visible. The periodicity is 5 ± 1 nm, in perfect agreement with the diffraction pattern.

Note that a fraction of the domain areas could be (1 × 1) rather than (2 × 1) without changing the diffraction pattern significantly. The only consequence would be a slight change in the relative peak intensities. An estimate for the surface corrugation within a domain of 0.07+/− 0.05 nm can be obtained from the diffraction pattern, using a hard wall model for the helium surface interaction and assuming that the diffraction pattern consists of three (2 × 1) domains with no (1 × 1) contribution.

So how can domains appear on a quartz surface? A suggestion for this was first presented in^11^. Here it is shown how steps of a height corresponding to 1/3 of a bulk unit cell (about 0.16 nm), create a surface with three domains rotated 120° relative to each other. The height difference between the domain areas in the AFM image in Fig. 2 is measured to be between 0.1 and 0.28 nm. This is the first measurement of an atomic step height on quartz and fits the model presented in^11^.

### Theoretical Calculations

Our early calculations^6^ confirmed a year later by Car *et al*.^7^ suggest a (1 × 1) reconstruction (referred to as the bridge structure or the dense structure) to be the absolute minimum energy surface for α-quartz (0001) and a (2 × 1) surface reconstruction (referred to as the zig-zag or semi-dense structure) to be very close in energy. A recent theoretical paper^3^ proposes a surface, with patches of (1 × 1) and (2 × 1) forming a large surface reconstruction of stripes. However this cannot explain the rotation seen in the experiments. Further the simulation is based on 10 bulk unit cells, smaller than the reconstruction we observe experimentally.

The results reported in^10^ together with our observations show that the surface reconstruction of α-quartz (0001) depends crucially on the thermal history of the crystal. To verify this, we performed a range of simulations. These included DFT simulations using the VASP code^21^,^22^, where we modelled slabs 2 nm thick containing typically 72 atoms. We used the PBE functional with a plane wave cut off of 500 eV, a K point grid of 4, 4, 1 and converged the forces to 0.001 eV/nm. The energies and structures from the DFT simulations were then used to verify the reliability of the BKS interatomic potential^23^. The advantage of the use of interatomic potentials is that much larger systems can be investigated. Thus we used the BKS potential within the LAMMPS code^24^ to model the surfaces of a range of different configurations annealed at different temperatures. Simulation cells started from around 8712 atoms, which again consisted of slabs approx. 2.0 nm (thickness: 4 unit cells) with different shaped surfaces extending up to simulation cells of 278784 atoms in order to allow structures with different periodicities to form. The annealing process comprised of a series of 100 ps simulations, each of which linearly scaled the temperature from 1 K to the specified temperature and back. Three temperatures were chosen and run successively, 10 K, 300 K and 1500 K. This procedure allows the surface to reconstruct at low temperatures and then anneal to the preferred configuration at higher temperatures

**Figure 3:**
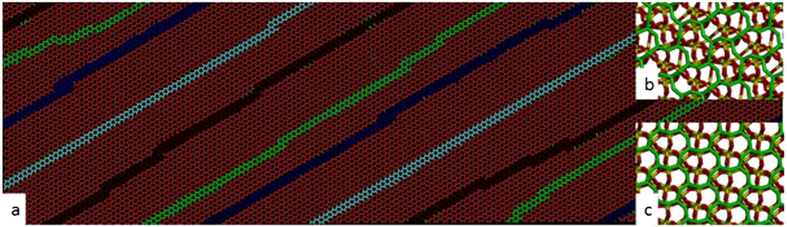
(**a**) Simulated annealed surface showing four stripes of the bridge reconstruction amongst the zig-zag reconstruction coloured dark blue, light blue, brown and green for clarity. The inset (**b,c**) show surface cells with the (2 × 1) zig-zag reconstruction and the (1 × 1) bridge reconstruction.

In Fig. 3(a) we show a snapshot from the 278784 atom simulation in which the (0001) surface was heated from 1 K to 300 K (below simulated melting temperature) for 50ps followed by cooling from 300 K to 1 K over a further 50ps. We do see four distinct stripes (coloured dark blue, light blue, brown and green for clarity) formed by (1 × 1) and (1 × 2) reconstructions, similar to^3^ and with a periodicity of around 5 nm matching the experimental results. However, the reconstruction is not rotated relative to the bulk unit cell. Furthermore, it would be difficult to see in the AFM because the stripes do not differ in height from the rest of the surface. Further examination reveals that the surface is largely the (2 × 1) reconstruction (Fig. 3b) while the stripes are (1 × 1) (Fig. 3a).

**Figure 4:**
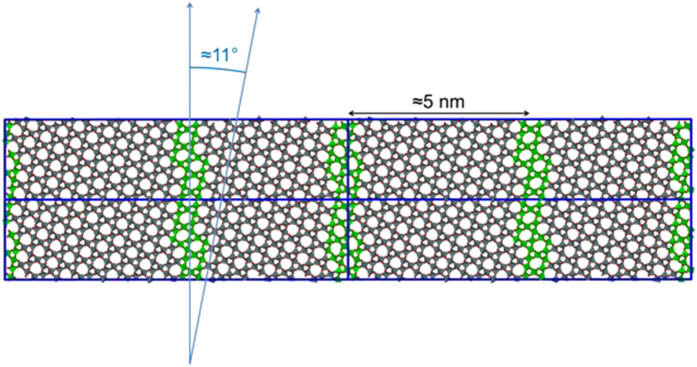
An unreconstructed surface comprised of domains of Dauphine twins, alternating with a period of about 5 nm between the boundaries (green). The twin boundaries are oriented in the 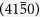 direction, corresponding to a rotation relative to the bulk unit cell of about 11°, in agreement with the HAS diffraction pattern.

Despite repeated annealing of the surface we did not find spontaneous formation of the large, experimentally observed rotated reconstruction during the simulations. One of the problems for simulation of silica in general is the many deep local minima, as demonstrated by the many polymorphs, the ease of glass formation and the slow crystallisation kinetics. Moreover, the highly energetically favourable reconstructions at the surface reduced the mobility near the surface and hence preventing the simulations exploring configurational space over the lifetime of the simulation. Thus we approached the problem by applying the annealing procedure on large bulk simulation cells, before the surface is cut. A number of different seed configurations and reconstructions were applied to bulk quartz, for example by partially melting but keeping different portions of the crystal fixed and by introducing different interfaces^25^. In particular we manually introduced Dauphine twins to test their stability. Dauphine twins in bulk quartz can be created during growth or produced from β-quartz by cooling through the inversion temperature of 573 °C^26^,^27^. The conversion from β to α-quartz can be described geometrically as a rotation around an angle θ in the (0001) plane. In β-quartz θ = 0°, but in α-quartz θ is either positive or negative 16.3°. This gives rise to two distinct α_1_ and α_2_ twin orientations. The chirality is not changed. One twin orientation can be transformed to the other by a rotation of 180° in the (0001) plane. For an excellent overview drawing see^28^. Dauphine twinning has been reported in natural quartz on a huge length scale range from macroscopic to microscopic^29^,^30^. In Fig. 4 we show a generated “stripe” configuration with a 5.1 nm period, corresponding to 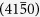 Dauphine twin boundaries, i.e. either side of the boundary has different Dauphine twins. In this simulation the twin boundaries have been rotated about 11° relative to the bulk unit cell and are dynamically stable at lower temperatures). However the twin does not survive at higher temperatures. Additionally, the direction and orientation of the Dauphine twin boundary is highly variable and dependent upon the size and orientation of the simulation cell being used. Therefore, we considered larger simulation cells where the direction and orientation of any spontaneously formed Dauphine boundary is less influenced by the existence of periodic boundary conditions.

A large bulk simulation cell (74160 atoms) of pure α-quartz was heated to 2000 K at constant volume and equilibrated for 10ps, at this temperature the material is β-quartz. The temperature was then reduced linearly from 2000 K to 100 K over the course of 20ps. As the temperature decreased different regions of the crystal relaxed rapidly to α-quartz and, importantly, independently of each other. As the relaxation is independent the orientation of the α-quartz regions do not necessarily match, and hence Dauphine twins may form (at slower cooling rates we observed annealing of these regions back into a pure cell of α-quartz). The structure was then energy minimised for 10000 steps in LAMMPS to remove thermal disorder from the structure.

**Figure 5:**
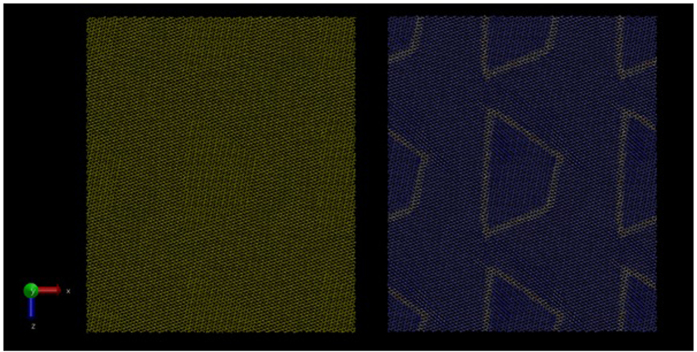
Final frame of the 74160 atom simulation (expanded 2 × 2x for clarity) showing a plan view of the (0001) surface before and after analysis to highlight the twin domain boundaries. The trapezoidal shape of the inner domain is reflective of the symmetry of quartz, but the domain itself is misaligned with the quartz crystal.

At this stage observing the formation of the Dauphine twins and the boundaries between them is very challenging due to the very small differences in atomic positions between the twins. We found a robust approach for locating them by calculating the atomic density within a 4.5 Å radius and colouring the atoms accordingly. Applying this approach to the 74160 atom simulation revealed the twin boundaries presented in Fig. 5.

The Dauphine twin region of the 74160 atom simulation had a shape reminiscent of a trapezoid, with two 60° angles and two 120° angles, indicating a relationship of the region geometry to the quartz structure underneath. However, the Dauphine region does not sit along the crystallographic directions of the quartz, but at an offset that does indeed vary around 11°. We discovered that these types of structures were easily annealed back to pure crystal at room temperature, but are stabilised by the presence of the surfaces. Further evidence that the domain structure may be “pinned” by the surface.

**Figure 6:**
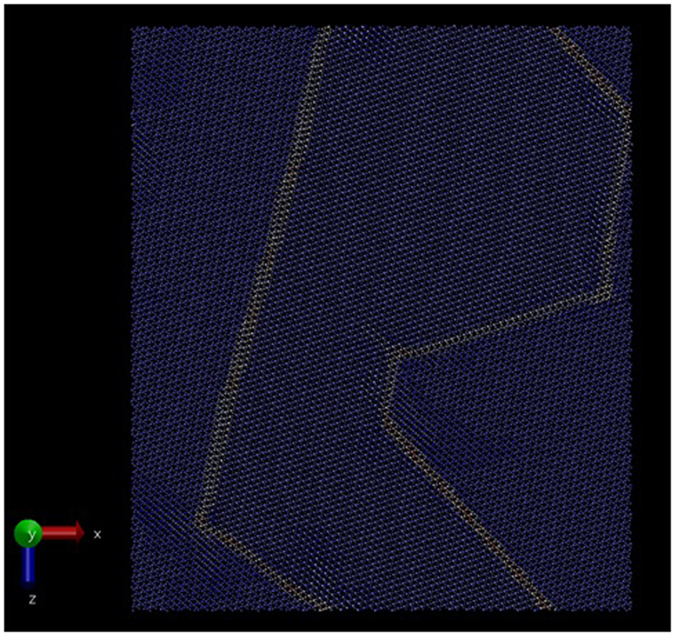
Final frame of 296640 atom simulation displaying abundance of 60° and 120° angles, but once again misaligned from the underlying lattice.

**Figure 7:**
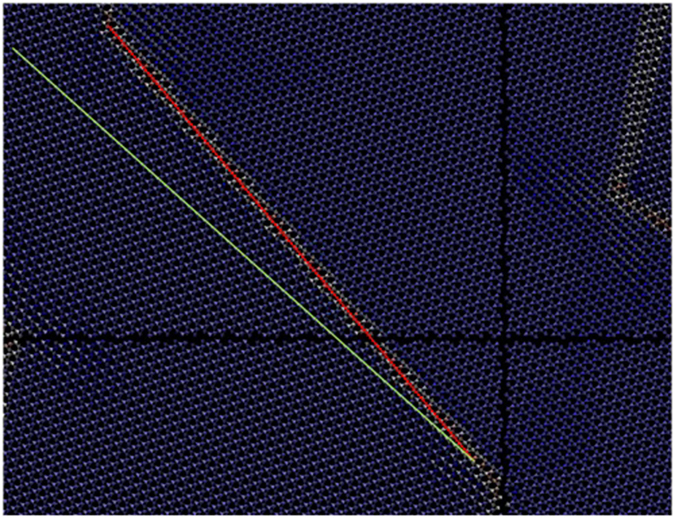
Detail of a boundary from Fig. 6. The green line follows a crystallographic direction while the red follows the boundary. The angle of mismatch here is approximately 10°. The bonds between the atoms of adjacent simulation cells are not drawn to show the location to show the size and positions of the periodic boundary.

To explore further the stability of the Dauphine twins we conducted an even larger simulation of 296640 atoms using the same methodology as before. Dauphine twin formation was once again observed to occur spontaneously with the characteristic 60° and 120° angles, once again misaligned from the underlying quartz lattice. Upon annealing at 300 K the twinning remained stable, with the smaller domains being absorbed or destroyed by the largest domains. The remaining structure showed Dauphine regions extending infinitely across the periodic boundaries, which may assist stability (Fig. 6). The angle of misalignment varied depending on the boundary segment chosen but always had some small misalignment; the segment shown in Fig. 7 displays the mismatch of the domain boundary and the lattice. Importantly the structure remained stable at 500 K, whereas the previous twin did not, indicating the importance of size effects in these simulations.

The stability of the Dauphine regions seems heavily dependent upon the inter-boundary distance and is also influenced by the simulation cell geometry. This can explain why the fine detail of the mismatch angle may not have been noticed by simulations before. Indeed, even observing the Dauphine regions required careful analysis of the final structure. The angle of mismatch itself appears to be the result of the crystal attempting to relieve the strain generated by the two Dauphine domains meeting because each domain would prefer the rotation of the silicate tetrahedra at the interface to be in opposite direction. The mismatch of the boundary and lattice is thus likely a result of the strain accumulating along a silicate chain which is then relieved periodically by shifting the strain to an adjacent chain. While the exact angle produced in these simulations may vary by 1° or 2° between models and simulation cells, the fact that these regions do not align along the crystallographic directions as may be expected, is very significant.

The simulations showed that on cooling from β-quartz that the different forms of α-quartz nucleate separately in the very large simulation cells and these give the domain boundaries where the grains meet. These boundaries show a characteristic angle with the crystallographic unit cell and the simulations suggest that the mismatch of the boundary and lattice is thus likely a result of the strain accumulating along a silicate chain which is then relieved periodically by shifting the strain to an adjacent chain. At the (0001) surface in particular can undergo a strong reconstruction, as we have shown before. The rebonding at the domain boundaries effectively pins the boundary.

## Discussion

LEED measurements published in^11^ reports a (√84 × √84) R11° diffraction pattern from an α-quartz (0001) surface that has been briefly heated above 600 °C and then cooled to room temperature. No error bars are provided and no atomic model, which can explain the diffraction pattern proposed. The rotation of the diffraction pattern in^10^ is similar to the diffraction pattern that we measure, but the periodicity is smaller. This could be due to the shorter annealing time compared to our samples. However it is also very possible that the smaller periodicity is simply an experimental artifact associated with charging effects and that the diffraction pattern that they observe is in fact identical to ours.

It is not possible on the basis of our measurements and simulations to say how deeply into the bulk, the reconstruction that we observe really goes. In principle the whole crystal could be reconstructed, but this is not likely. Firstly, our measurements clearly show that the reconstruction display a domain structure, which follows the atomic steps on the surface. This indicates that the phase transformation starts at the surface, similar to a premelting process^31^. Secondly the reconstruction starts to form already after a brief annealing as described in^10^. Finally no similar reconstruction has been found when thin quartz samples have been heated and investigated in TEM. References^32^,^33^ report an intermediate phase between α and β-quartz, consisting of triangular nano Dauphine twins, which is stable over a 1.3 degree temperature interval close to the transition temperature, but not at room temperature. It is reported that the Dauphine twin structures are never periodic over a long range, though it is speculated that this may be due to a strong temperature gradient in the sample during the TEM investigation. According to^33^ the Dauphin twin size increase with decreasing temperature from 10 nm up to 100 nm and greater. Reference. 27 reports Dauphine twins in the nano/micron size-range on TEM α-quartz (0001) samples that have been heated above the transition temperature and then cooled to room temperature, but they too do not have a long range periodic structure. Thus we suggest that it is the presence of the thick bulk crystal which “fixes” the Dauphine nano-twins in a long range periodic reconstruction of the top surface layer(s).

Dauphine twinning of quartz is associated with a change of the piezoelectric constant^34^. Recently ultrafast switching in piezoelectric thin films with nanodomains^35^ and the controlled growth of α-quartz thin films on a silicon surface^36^ were demonstrated. The Dauphine nanotwin surface reconstruction could potentially be exploited to create tailored nanostructured piezoelectric materials with novel properties.

Our results further indicate that surface investigations of natural quartz crystals can reveal new information about their geological/geophysical history and properties, ie. changed absorption properties due to surface reconstructions caused by heating above a certain temperature. Finally we suggest that nano-scale reconstructions of quartz may contribute to explain the illness silicosis, which occurs upon inhaling crystalline silica dust in the micron range, but not upon inhaling glass dust of similar particle size^37^. Silicosis is essentially an inflammatory response of the body immune system to the dust. It has been proposed that this is caused by plasma proteins being deformed upon adsorption on the quartz surface so that they are seen as “alien” by the immune system^38^. It is plausible that surface reconstructions with periodicity on the nano-scale could provide suitable absorption sites and influence the folding of proteins.

## Methods

HAS is not a commercially available technique. Our HAS apparatus has a unique angular resolution of 0.001°, more than one order of magnitude better than all other instruments^39^. The AFM measurements were performed with a IIA Nanoscope, Digital Instruments Inc., using 40 N Si_3_N_4_ cantilevers from Nanosensors.

10 mm × 10 mm × 1 mm samples were prepared from a synthetically grown, twin free α-quartz crystal cut along the (0001) direction with a miscut of less than 0.5°. Samples were mechanically polished to optical quality using diamond powder and annealed to nominally 1025 °C in a furnace for 72 hours in a 2 bar oxygen atmosphere at Saint-Gobain Recherche. The heating rate was 5 K/min up to 300 °C with a dwell at 300 °C for two hours, followed by a heating to the final temperature at a rate of 10 K/min. The cooling down rate was 3 K/min down to 750 °C, then 5 K/min down to 500 °C. After this the cooling rate was free (the power was cut). The furnace setting was determined by a thermocouple placed next to the sample. For this reason the actual annealing temperature of the sample is estimated to be up to 20 K lower than the nominal annealing temperature (1025 °C). Two samples were prepared in parallel. One was used for AFM studies, the other was transported and mounted in the HAS apparatus under Argon. After an initial oxygen cleaning, described in^11^ the sample maintained its room temperature reflectivity over a period of more than two months in a vacuum with a base pressure in the low 10^−8^ mbar range. Measurements were performed on 5 samples, showing that the structure is very reproducible.

For the HAS experiments a monochromatic beam of helium atoms was created by supersonic expansion from a 10 μm diameter nozzle. A skimmer, 410 μm in diameter, selected the central part of the beam. The beam was detected using a home built electron bombardment detector. The distance between the skimmer and the sample was 1528 mm and the distance between the sample and the detector 942 mm. The beam was nitrogen cooled to 25.2+/− 0.1 meV (corresponding to λ = 0.096 nm) and had an energy spread ∆E/E of about 1.6%. To increase the reflectivity the sample was cooled down to less than 150 K during measurements. The angle between the source and the sample was kept fixed at 45°. The detector was rotated in steps of 0.005° corresponding to a ∆k of 0.014 Å^−1^.

## Additional Information

**How to cite this article**: Eder, S. D. *et al*. A Giant Reconstruction of α-quartz (0001) Interpreted as Three Domains of Nano Dauphine Twins. *Sci. Rep*. **5**, 14545; doi: 10.1038/srep14545 (2015).
